# Awareness of sex and gender dimensions among physicians: the European federation of internal medicine assessment of gender differences in Europe (EFIM-IMAGINE) survey

**DOI:** 10.1007/s11739-022-02951-9

**Published:** 2022-05-23

**Authors:** Ewelina Biskup, Alberto M. Marra, Immacolata Ambrosino, Elena Barbagelata, Stefania Basili, Jacqueline de Graaf, Asunción Gonzalvez-Gasch, Risto Kaaja, Eleni Karlafti, Dor Lotan, Alexandra Kautzky-Willer, Maria Perticone, Cecilia Politi, Karin Schenck-Gustafsson, Andreia Vilas-Boas, Jeanine Roeters van Lennep, Emma A. Gans, Vera Regitz-Zagrosek, Louise Pilote, Marco Proietti, Valeria Raparelli, Internal Medicine Assessment of Gender differences IN Europe (IMAGINE) Working group within the European Federation of Internal Medicine (EFIM), Nicola Montano, Runòlfur Pàllson, Valentin Korokin, Xavier Corbella, Daniel Sereni, Rijk Gans

**Affiliations:** 1 Division of Internal Medicine, University Hospital of Basel, Basel, Switzerland; 2 Department of Translational Medical Sciences, “Federico II” University of Naples, Naples, Italy; 3 Geriatrics, Local Healthcare Unit of Bari, Health District 13, Bari, Italy; 4 Department of Internal Medicine, Lavagna Hospital ASL 4 Chiavarese, Genoa, Italy; 5 Department of Translational and Precision Medicine, Sapienza University of Rome, Rome, Italy; 6 Radboud University Medical Centre, Radboud Health Academy - division of PGME, Nijmegen, Netherlands; 7 Unidad de Medicina Interna, Hospital Quirónsalud Torrevieja, Alicante, Spain; 8 Internal Medicine, University of Turku, Turku, Finland; 9 1st Propedeutic Clinic of Internal Medicine, AHEPA University Hospital of Thessaloniki, Thessaloniki, Greece; 10 Division of Cardiology, Columbia University Irving Medical Center, New York, USA; 11 Gender Medicine Unit, Division of Endocrinology and Metabolism, Department of Internal MedicineIII, Medical University Vienna, Vienna, Austria; 12 Department of Medical and Surgical Sciences, Magna Graecia University of Catanzaro, Catanzaro, Italy; 13 Internal Medicine, “F. Veneziale” Hospital, Isernia, Italy; 14 Centre for Gender Medicine, Department of Medicine, Karolinska Institutet and Karolinska University Hospital, Stockholm, Sweden; 15 Internal Medicine, Hospital da Luz Arrábida, Vila Nova de Gaia, Portugal; 16 Department of Internal Medicine, Erasmus MC University Medical Centre, Rotterdam, Netherlands; 17 Department of Internal Medicine, Leiden University Medical Center, Leiden, Netherlands; 18 Charité, University Medicine Berlin, DZHK, Berlin, Germany; 19 Division of Clinical Epidemiology and General Internal Medicine, McGill University Health Centre Research Institute, Montreal, Canada; 20 Department of Clinical Sciences and Community Health, University of Milan, Milan, Italy; 21 Geriatric Unit, IRCCS Istituti Clinici Scientifici Maugeri, Milan, Italy; 22 Liverpool Centre for Cardiovascular Science, University of Liverpool and Liverpool Heart and Chest Hospital, Liverpool, UK; 23 Department of Translational Medicine, University of Ferrara, Via dei Borsari 46, 44121 Ferrara, Italy; 24 University Center for Studies On Gender Medicine, University of Ferrara, Ferrara, Italy; 25 Faculty of Nursing, University of Alberta, Edmonton, Canada; 26 Department of Advanced Biomedical Sciences, Federico II University of Naples, Naples, Italy; 27 Center for Pulmonary Hypertension, Thoraxklinic, University Hospital Heidelberg, Member of the German Center for Lung Research (DZL), Heidelberg, Germany; 28 University of Zurich, Zurich, Switzerland; 29 Gender Institute, Gars am Kamp, Austria

**Keywords:** Sociocultural gender, Biological sex, Personalized medicine, Internal medicine, Education

## Abstract

Sociocultural gender is a complex construct encompassing different aspects of individuals’ life, whereas sex refers to biological factors. These terms are often misused, although they impact differently on individuals’ health. Recognizing the role of sex and gender on health status is fundamental in the pursuit of a personalized medicine. Aim of the current study was to investigate the awareness in approaching clinical and research questions on the impact of sex and gender on health among European internists. Clinicians affiliated with the European Federation of Internal Medicine from 33 countries participated to the study on a voluntary basis between January 1st, 2018 and July 31st, 2019. Internists’ awareness and knowledge on sex and gender issues in clinical medicine were measured by an online anonymized 7-item survey. A total of 1323 European internists responded to the survey of which 57% were women, mostly young or middle-aged (78%), and practicing in public general medicine services (74.5%). The majority (79%) recognized that sex and gender are not interchangeable terms, though a wide discrepancy exists on what clinicians think sex and gender concepts incorporate. Biological sex and sociocultural gender were recognized as determinants of health mainly in cardiovascular and autoimmune/rheumatic diseases. Up to 80% of respondents acknowledged the low participation of female individuals in trials and more than 60% the lack of sex-specific clinical guidelines. Internists also express the willingness of getting more knowledge on the impact of sex and gender in cerebrovascular/cognitive and inflammatory bowel diseases. Biological sex and sociocultural gender are factors influencing health and disease. Although awareness and knowledge remain suboptimal across European internists, most acknowledge the underrepresentation of female subjects in trials, the lack of sex-specific guidelines and the need of being more informed on sex and gender-based differences in diseases.

**Supplementary Information:**

The online version contains supplementary material available at 10.1007/s11739-022-02951-9.

## Introduction

Recently, physicians have been witnessing a paradigm shift in clinical practice from a disease-centered to a patient-centered approach in the pursuit of personalized medicine [1]. The concept of sociocultural gender, as opposed to biological sex, emerged and gained prominence in all domains of life, including health and disease [2–4]. It became apparent that sociocultural gender, encompassing different aspects of individuals life including identity, role, relations and institutionalized gender, has an impact in shaping the health of individuals at least equal or even beyond the biological sex. The promotion of these concepts in clinical medicine is pivotal to build personalized and tailored approaches for prevention, diagnostics, therapeutics and disease management [5, 6]. The intersectionality of sex and gender is an important yet neglected determinant of health which deserves attention. [7, 8] Accordingly, the incorporation of a sex (i.e. biological factors) and gender (i.e. psycho-socio-cultural factors) lens in clinical research and practice is promoted internationally as a promising strategy to better science and to improve clinical and patient-relevant outcomes [9, 10]. A solid awareness in regards to sex and gender being essential biomarkers and influencing parameters in health- and seeking care, is a cornerstone towards adopting sex- and gender-informed decisions in clinical practice. To achieve such awareness, the first step is to provide extensive evidence on their impact on diseases through clinical studies. A first obstacle clinicians have to face is that women are commonly underrepresented in clinical trials with a lack of sex-disaggregated data, thus the feasibility of sex-specific clinical decision-making is limited [5, 8]. The assessment of gender domains is even more challenging as there is no standardized measure of gender. Nevertheless, when gender-related factors are collected, sociocultural gender is able to predict outcomes to a greater extent than biological sex alone [10].

Given the magnitude of sex and gender effects on health and disease, that has been recently reviewed elsewhere [5, 11] (Table 1), it is of utmost importance to assess the level of knowledge about the sex and gender dimensions among physicians to better identify areas where improvements can be pursued through interventions such as tailored education. Therefore, the aim of this study was to systematically investigate the knowledge and awareness of the internal medicine community in Europe on sex and gender dimensions in approaching clinical and research questions.

**Table 1:** Examples of how sex and gender influence on health and diseases[5, 11]

Area	Example physiology/pathology	Sex-based considerations	Gender-based considerations
Genetics	X chromosome. Y chromosome. Parental Imprinting.	Sex differences in gene regulation due to X chromosome inactivation and autosomal-like regions contained in the X chromosome	–
Epigenetics	DNA methylation. Histone modification. Gene silencing by noncoding RNA	Sex dimorphism and genetic variation driven by endogenous sex-specific hormones	Diet, exercise, cigarette smoking, environmental toxins, and psychosocial stress modify gene expression.
Pharmacology	Pharmacodynamics Pharmacokinetics	Sex differences in absorption, distribution, metabolism, and elimination of pharmacological agents. Higher incidence of adverse drug reactions in females.	Gender-related factors (e.g., greater caregiving responsibilities, trauma exposures) influence the efficacy of behavioral treatments of addiction in women and transgender individuals. Medication adherence can be influenced by gender-related factors.
Cardiovascular system	Cardiovascular diseases	Prevalence, clinical manifestations and outcomes, and response to main cardiovascular therapies differ by sex (e.g., obstructive vs non-obstructive coronary artery disease; higher mortality in young females with acute myocardial infarction). Sex-specific risk factors (e.g., eclampsia, gestational diabetes). A disproportionate percentage of stroke deaths in females.	Exposure to detrimental social determinants of health (such as poverty, low education, and health literacy, insurance coverage) influences cardiovascular outcomes. Treatment disparities due to insufficient recognition of different symptomatology
Liver function	Liver function	Sex-differences in disease susceptibility (higher risk of primary sclerosing cholangitis, chronic viral hepatitis, cirrhosis, and hepatocellular carcinoma in males; higher risk of primary biliary cholangitis and autoimmune hepatitis in females). Sex-based response to therapies (non-alcoholic steatohepatitis resolution with moderate body weight loss for males whereas much greater weight reduction in females is needed).	Women follow healthier diets by eating more vegetables and fruits and less meat and fat than men so that they are less at risk of non-alcoholic fatty liver disease
Kidney function	Chronic kidney diseases	Higher prevalence in females; sex-specific risk (hypertensive disorders of pregnancy). More rapid rate of progression in the impairment of renal function in males.	Gender biases in renal transplant allocation
Respiratory system	Chronic obstructive pulmonary diseases (COPD) asthma	In females, early onset of COPD with less tobacco exposure and higher exacerbation rates than males. Higher prevalence of asthma in middle-aged females. More likely premenstrual asthma that improves after menopause	Gender-based change in smoking habits Gendered stereotypes driving misdiagnosis in women
Immune function	Autoimmune disorders	Sex dimorphism in prevalence of autoimmune diseases (innate and adaptive immune responses are stronger (e.g., higher vaccine efficacy) in females than males.	Gender-specific environmental exposures can induce vitamin D levels differences
Neurocognitive aging process	Alzheimer disease	Prevalence and rapid progression of cognitive impairment in females. Sex-specific effect in carriers of the APOE*E4 allele.	Gender-specific behavioral and lifestyle factors (e.g., smoking, regular physical activity) significantly influence brain aging. Greater burden of disease caregiving in women.
Mental health	Depression	Symptomatology partially different. Women have a higher predisposition to depression.	Men do seek treatment less than women but when they do, they are less likely to be diagnosed with major depressive disorder regardless of their scores on standardized measures of depression like those of women.
Intersection with healthcare system	Accessibility/barriers to care	Sex differences in the burden of several diseases requiring hospitalization or access to outpatient services.	Discrimination related to gender identity. Financial and non-financial (e.g., caregiving, transportation) barriers to accessing health care and treatment.

## Methods

The European Federation of Internal Medicine (EFIM)–the largest scientific society of Internal Medicine in Europe–has created a dedicated working group to approach this issue: the Internal Medicine Assessment of Gender differences in Europe (IMAGINE) working group [12].The design and the goals of IMAGINE working group have been previously published [12]. The first objective of IMAGINE was to verify the awareness of sex and gender-related differences on a random sample of resident or specialized internists in Europe. For this reason, a short anonymous online survey was administered to clinicians affiliated with EFIM.

### The IMAGINE survey

The IMAGINE survey was designed as an online short questionnaire to understand how the sex and gender dimensions are considered and perceived among internists. The study was performed in line with the principles of the Declaration of Helsinki. The study was exempted by Ethic Committee approval because of the anonymous nature of the survey. Participants provided their online written informed consent before filling in the survey.

The short online multiple-choice survey was composed of seven questions (Supplemental Fig. 1). Briefly, the first 3 questions aimed at assessing the knowledge on terminology (i.e. sex vs. gender) and the awareness of factors specifically related or not to sex and gender dimensions. The fourth question explored the perceived knowledge on sex and gender differences in major diseases within the field of internal medicine. The fifth and sixth questions sought to identify if physicians usually look in clinical guidelines for the presence of recommendations specifically tailored according to sex and whether they are aware of the low rate of women’s enrolment in clinical trials. Finally, the seventh question targeted the identification of high-priority topics for the internal medicine community in terms of willingness to acquire knowledge from sex- and gender perspective. The questionnaire was transferred on a freely available digital platform and circulated as an electronic link through an e-mail distribution list, as detailed below.

### Survey target population and dissemination strategy

The survey was circulated strategically among all members of EFIM between January 1st 2018 and July 31st 2019. Briefly, EFIM encompasses 35 internal medicine societies among 33 countries. The respective IMAGINE WG member (representative of each country) was also the national coordinator of the study and responsible for the country-specific dissemination of the survey via their national society network, via direct links to the hospitals or via hospital representatives. In addition, professional social media networks of the EFIM were used to popularize the survey. All professionals had to give mandatory information such as (1) age (years) and sex; (i2) practicing country; (3) professional position status; and (4) specialty/subspecialty and years of practice. The invitation was repeated at least 3 times during the recruiting period.

### Statistical analysis

The average of EFIM active members is 2000 (average of annual congress attendee), the minimum random sample size computed for the present survey would be 1200 subjects, considering a margin error of 5% and a confidence level of 95% and a response distribution of 50%. Random sample of surveyed participants was representative of EFIM-affiliated Residents + Specialists in IM. The Imagine Survey was launched during the 2018 EFIM congress in Wiesbaden (Germany).

Continuous variables were expressed as mean and standard deviation (SD) and differences between groups were evaluated according to the Student’s *t* test or ANOVA test, as appropriate. Categorical variables were expressed as counts and percentages. Differences among the different groups were evaluated by chi-square test. A logistic regression analysis was performed to identify those baseline characteristics associated with the ‘Yes/I Don’t Know’ answer to the question ‘Do you think that the terms “Sex” and “Gender” are synonymous?’. After univariate analysis, all those baseline characteristics significantly associated with the answer were included in a multivariable model. Additionally, we also performed a subgroup analysis about male and female respondents regarding question one and question two of the survey. A two-sided *p* value < 0.05 was considered to be statistically significant. All analyses were performed using SPSS v. 25.0 (IBM, NY, USA).

## Results

### Baseline characteristics of survey responders

The baseline characteristics of the participants are illustrated in Table 2. Overall, 1,323 individuals participated in the survey, with a similar distribution of females (56.9%) and males (42.6%), with a mean (SD) age of 42.3 (12.6) years. The predominant group was that of young age (51.2%, aged less than 39 years), from Western and Southern Europe (over 90%) and engaged as internal medicine health care specialists (85.1%), rather than other specialists or clinical scientists (8%). The mean (SD) amount of practicing years was 15.3 (12.5), with a very similar distribution in categories from 0–4 to 20–39 years (each ~ 20%).

**Table 2:** Baseline characteristics of survey’s participants

	Survey cohort *N*=1323	OR (95% CI)	*p*
Sex, *n* (%)			
Male	564 (42.6)	1.48 (0.17–12.81)	0.72
Female	753 (56.9)	1.27 (0.15–10.90)	0.83
Other	6 (0.5)	Ref.	Ref.
Age, years mean (SD)	42.3 (12.6)	0.99 (0.98–1.01)	0.40
Age classes, *n* (%)			
<29	180 (13.6)	0.91 (0.33–2.48)	0.86
30–39	498 (37.6)	1.29 (0.51–3.29)	0.59
40–49	252 (19.0)	1.64 (0.65–4.14)	0.29
50–59	232 (17.5)	1.31 (0.53–3.23)	0.56
60–69	126 (9.5)	1.25 (0.48–3.23)	0.64
≥70	35 (2.6)	Ref.	Ref.
European region, *n* (%)			
Northern Europe	34 (2.6)	0.88 (0.24–3.24)	0.85
Western Europe	578 (43.7)	2.29 (0.51–10.28)	0.28
Eastern Europe	27 (2.0)	0.84 (0.23–3.09)	0.79
Southern Europe	670 (50.7)	1.82 (0.42–7.90)	0.42
Non-EU countries	13 (1.0)	Ref.	Ref.
Work setting, *n* (%)			
General/primary care	1,126 (85.1)	Ref.	Ref.
Specialized care	77 (5.8)	0.89 (0.50–1.59)	0.69
Research centre	29 (2.2)	–	–
Other	91 (6.9)	1.47 (0.91–2.37)	0.11
Type of practice, *n* (%)			
Public	989 (74.8)	1.28 (0.70–2.35)	0.42
Private	37 (2.8)	0.81 (0.29–2.22)	0.68
Public and private	204 (15.4)	1.12 (0.66–1.92)	0.67
Other	93 (7.0)	Ref.	Ref.
Role, *n* (%)			
Junior physician	424 (32.0)	0.90 (0.47–1.75)	0.76
Attending physician/GP	595 (45.0)	1.17 (0.65–2.13)	0.60
Senior physician	226 (17.1)	1.25 (0.68–2.29)	0.48
Other	78 (5.9)	Ref.	
Specialty, *n* (%)			
Internal medicine/geriatric	803 (60.7)	0.86 (0.61–1.21)	0.37
Intensive care	33 (2.5)	1.04 (0.72–1.50)	0.82
Clinical sub-specialties	210 (15.9)	1.16 (0.51–2.61)	0.73
Physician in training/other	277 (20.9)	Ref.	
Practice years, mean (SD)*	15.3 (12.5)	0.99 (0.98–1.00)	0.22
Practice years, *n* (%)*			
0–4	292 (22.1)	Ref.	Ref.
5–9	293 (22.1)	0.79 (0.53–1.18)	0.26
10–19	290 (21.9)	1.05 (0.71–1.54)	0.81
20–39	365 (27.6)	0.87 (0.60–1.26)	0.46
≥40	76 (5.7)	0.57 (0.28–1.14)	0.11

*GP* General Practitioner, *SD*  Standard Deviation

*Data available in 1316 participants

### Gender and sex knowledge among  IMAGINE responders

The first question approached the general knowledge about the terms sex and gender and whether these are synonymous. Almost 79% of the surveyed individuals responded correctly (no), however as much as 15% responded incorrectly (yes) and almost 6% did not know the answer.

Regarding the association between the baseline characteristics and the answer ‘Yes/I Don’t Know’ to the first question, the univariate analysis found that no characteristic was significantly associated with the answer (Table 2). Hence, the multivariable model was not compiled.

**Fig. 1:**
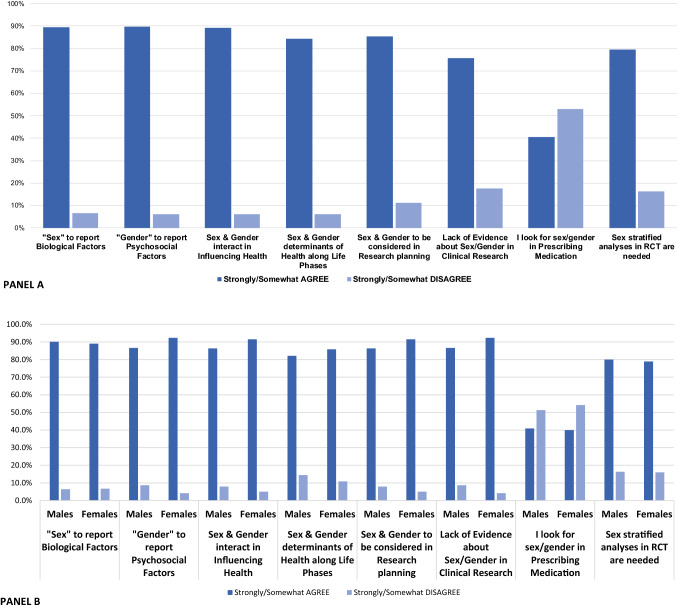
Degree of agreement and disagreement on sex and gender-based statements in the overall cohort (Panel 1A) and stratified by sex (Panel 1B)

Most participants agreed or strongly agreed on statements assessing the general knowledge of sex vs. gender (Fig. 1A), especially over 90% agreed that sex relates to biological factors, gender to psychosocial factors, both are interactively influencing health and their stratification is needed in research planning and in randomized controlled trials. On the other hand, less than half of the respondents (40%) consider sex and gender while prescribing medications in daily practice.

The analysis stratified according to the biological sex of responders showed that, as compared with male responders, female responders were more likely to strongly agree about: the significance of the term “gender” (58.0 vs. 54.3%, for females and male respectively; *p* < 0.001); the interaction between sex and gender in influencing health (55.2 vs. 50.5%, respectively; *p* = 0.05); the role of sex and gender as determinants along with life phases (45.3 vs. 40.8%; *p* = 0.05); considering sex and gender in the research planning (46.9 vs. 42.6%; *p* = 0.021); and that there is a lack of evidence in clinical research about differences between sex and gender (42.8 vs. 29.8%; *p* < 0.001) (Fig. 1B).

### Identification of sex and gender-related factors

On average, 50–60% of the participants correctly allocated sex as a determinant of body size, genetics, sex hormones, reproductive status and body composition. Only 30–40% correctly allocated gender as a determinant of diet, personality traits, marital and socio-economic and working status. As much as 50–60% stated that ethnicity, religion, age, comorbidities, disabilities, environment and geographic location are neither sex nor gender related (Table 3).

**Table 3:** Identification of sex and gender-related factors among the overall cohort of surveyed participants

	Sex related (%)	Gender related (%)	Sex and gender related (%)	No sex and gender related (%)	Don’t know (%)
Body size	49.9	15.0	14.6	17.9	2.6
Genetics	66.4	11.5	12.8	6.0	3.4
Sex hormones	66.1	11.7	18.7	1.5	1.9
Reproductive status	64.0	12.9	16.9	3.5	2.7
Body composition	54.0	18.8	20.2	4.8	2.2
Diet	8.3	32.9	14.2	40.1	4.5
Marital status	10.6	41.6	12.9	27.6	7.3
Personality traits	6.8	44.7	21.1	20.6	6.8
Socio-economic status	6.9	35.4	17.6	35.4	4.7
Working status	8.9	38.8	17.2	31.6	3.6
Alcohol	9.9	34.8	12.2	39.6	3.4
Smoking habit	8.0	34.0	11.3	42.2	4.5
Ethnicity	8.2	20.6	5.1	59.9	6.2
Religion	2.2	22.3	4.2	64.9	6.4
Age	22.4	10.4	8.2	54.9	4.2
Comorbidities	33.1	18.9	21.8	22.1	4.1
Disability	7.1	13.5	6.3	63.7	9.3
Geographic Location	4.1	16.0	3.9	66.6	9.4
Environment	4.3	30.7	8.8	45.7	10.5

### Beliefs and knowledge interest on sex and gender as determinants of health and diseases

Cardiovascular (92.9%), vascular (88.4%), lung diseases (70.9%), and inflammatory bowel disease (68.9%) were most frequently identified as being influenced strongly by sex and gender, while infectious (46.3%), renal (44.1%) and neuropsychiatric diseases (28.0%) were strongly misidentified as not being influenced by sex or gender. The areas where most participants expressed interest in learning more about sex and gender influence for application to their clinical practice were: cardiovascular (57.4%), vascular (16.7%), inflammatory bowel (6.7%) and neuropsychiatric (6.1%) diseases.

### Female participation in randomized clinical trials for approval of new drugs

Among participants, 41% responded that the current female representation in clinical trials ranges from 10 to 30% while 44% stated a mean proportion of 50%. Only 64% ever heard about specific recommendations for female individuals in guidelines.

### Discussion

The main result of the IMAGINE survey is that one-fifth of European residents or specialized Internists use the two terms “sex” and “gender” interchangeably. Even though most physicians stated that they are aware of the influence played by sex and gender in healthcare practice and research planning, they were not able to correctly identify what these concepts represent. Consequently, they do not actively apply that knowledge when it comes for example to medical prescriptions. This situation may be a consequence of the underrepresentation of women in randomized controlled trials for approval of new drugs and of the lack of specific sex/gender-driven guidelines. Indeed, European internists were aware of the lack of evidence. Strikingly it is acknowledged by a large pool of physicians that cardiovascular diseases are strongly influenced by sex and gender, whereas other pathological entities are not.

While biological sex might be primarily seen as determining health differences between males and females, gender is primarily implicated in inequalities resulting from varying patterns of social roles, behavior, attitudes and even lifestyles [11, 13]. Both sex and gender are influenced by diverse socio-cultural factors and social stratification, but gender is related to conditioning and stereotyping of personality traits considered typically “feminine” or “masculine”, which in turn significantly affect somatic and psychological well-being. Several studies confirmed that there are substantial biological differences between males and females, which drive various diseases and even brain aging, which further influences the development of a broad spectrum of diseases and shapes the attitude towards health status [14, 15]. Meanwhile, most guidelines are based on a pre-selected cohort of mostly male representatives (female participation in large randomized controlled trials is as low as 10–30% depending on the health domain) [16, 17]. Furthermore, there is a growing base of reports about major sex differences in pharmacology [18, 19]. Our survey is the largest to date and the first European study on sex and gender awareness, knowledge, interest and practice among internal medicine professionals, both trainees and advanced specialists. A specific lack in training and dissemination of the sex/gender issue is a knowledge gap urgently in need to be filled. Therefore, multidimensional gender-based frameworks across all diseases are essential for healthcare professionals. A significant raise in the awareness of the importance of sex and gender as core parameters influencing patients’ management both in prevention and in therapies, is needed and thus should be primarily perpetuated by scientific literature.

Gender as psycho-socio-cultural construct is still underestimated in its effect on the incidence, etiology, and development of diseases and the effectiveness of therapy in everyday clinical practice, given the differentiating impact of various external factors and reactivity of (epi)genome on health [22]. Clearly, this encompasses more than only cardiovascular diseases and endocrinology, but perhaps even more so respiratory diseases, gastro-hepato-enterology, hematology, neurology and autoimmune diseases. This also refers to sex- and gender-dependent differences in drug response, medication adherence and metabolism [23]. Finally, there is currently enough evidence that not only biological sex and age, but also sociocultural gender is an independent risk factor for individual diseases, and can largely determine their course. [9, 24].

Another finding of interest is that the intersectionality between sex, gender and other relevant features of individuals requires to be addressed and discussed with the internists’ community. Intersectionality involves the study of the ways that race, gender, disability, sexuality, class, age, and other social categories are mutually shaped and interrelated [9]. Information pertaining to how these other social constructs may interact with sex and gender to influence the risk of diseases and their clinical progression are warranted to guide internists in providing patient-individualized pathways of care.

### Clinical implications

Despite increasing evidence that an individual’s sex is one the most important modulators of disease risk and response to treatment, consideration of the patient’s sex in clinical decision-making is often lacking [25, 26]. This is a matter of concern as precision medicine, the new paradigm of the twenty-first century, should begin with attention to sex and gender differences. Surprisingly, there is a reduced awareness of biological, physiological and epidemiological differences between the biology behind medicine in men and women. In our survey, we spotlighted how the clinicians’ community is aware of the low participation of women in randomized controlled trials for testing new drugs and of the drawbacks of clinical guidelines that do not provide sex-specific recommendations.

Internal medicine is one of the core medical specialties. Internists as they are expected to manage and triage their patients to further, more narrow fields of medicine, need to be informed and trained on the latest of medical progress. As internists’ responsibilities are particularly related to complex clinical scenarios and disease patterns (e.g. multimorbidity, senescence, etc.), they should necessarily be familiar with the gender concept and carefully discern its categorically scientific elements and apply it in patient care. [27, 28].

While cardiovascular, neuropsychiatric and immune-endocrine fields have made tremendous advancements in integrating sex and gender in respective research focus, internal medicine is still relatively far from such progress due to the complexity of the field [12]. The IMAGINE WG thus aimed to explore the respective sex vs. gender knowledge and attitudes of the European internal medicine community, both in training and at an advanced career stage, conducting the largest survey in Europe thus far.

In conclusion, even if most internal medicine physicians are aware of the distinct nature of biological sex and sociocultural gender–they still experience difficulties in identifying what a gender-related variable is. Internists are largely conscious of female underrepresentation in trials and the lack of sex-specific guidelines in their field of interest. While the sex and gender impact on cardiovascular disease is quite well-recognized, expanding knowledge to address sex and gender-specific aspect across a larger spectrum of diseases is acknowledged. Areas of interest include cerebrovascular and inflammatory bowel diseases. The data collected through the IMAGINE survey can help to identify areas of improvement and the knowledge gaps about sex and gender to tackle among the European internal medicine clinicians.

## Supplementary Information

Below is the link to the electronic supplementary material.

Supplementary file1 (PDF 318 KB)

## Abbreviations

EFIM: European federation of internal medicine
IMAGINE WG: Internal medicine assessment of gender differences in europe working group
SD: Standard deviation
