# Supplementary material for: Awareness of sex and gender dimensions among physicians: the European federation of internal medicine assessment of gender differences in Europe (EFIM-IMAGINE) survey
Source: Intern Emerg Med. 2022 May 23;17(5):1395–404. doi: 10.1007/s11739-022-02951-9 (PMC9352607; doi:10.1007/s11739-022-02951-9)
Supplement: Supplementary file 1 — Supplementary file1 (PDF 318 KB) [file 11739_2022_2951_MOESM1_ESM.pdf]

Supplemental Figure 1

IMAGINE SURVEY

Sex/Gender awareness

IMAGINE
Internal Medicine and Assessment of Gender differences in Europe

Do you think that the terms "SEX" and "GENDER" are synonymous? *

- Yes
- No
- I do not know

Please, read the following statements, and indicate how much you agree or disagree with each one of them *

	Strongly agree	Somewhat agree	Somewhat disagree	Strongly disagree	Don't know	Decline to answer
The term "sex" should be used when reporting biological factors						
The term "gender" should be used when reporting gender identity or psychosocial or cultural factors						
Sex and gender can interact in influencing health disease						
Sex and gender are determinants of health along all life phases						
Sex and gender should be considered in research planning to personalize the management of disease						
There is lack of evidence exploring sex and gender differences in clinical research						
I always look for sex and gender specific information when prescribing medication						
In clinical trials a sex stratified-analysis should be always planned						

We are interested in knowing your opinion on how much awareness on sex and gender differences in health and disease and their management exist in your country. Please, read the following categories of health disease, and indicate the ones that you believe are influenced by sex and gender in your environment. *

	Strongly agree	Somewhat agree	Somewhat disagree	Strongly disagree	I do not know	Decline to answer
Cardiovascular Diseases						
Vascular disease other than cardiac ones						
Intestinal Bowel Diseases						
Kidney Diseases						
Cerebral Disease/Cognitive Diseases						
Lung Diseases						
Infectious Diseases						
Autoimmune Diseases						
Rheumatic Diseases						
Liver Diseases						
Blood Diseases						
Metabolic Diseases						
Mental disorders						
General use of drugs and medications						

Please for the following variables select the most appropriate condition (tick the ones you think are sex-related, gender-related or both or not?) *

	Sex Related	Gender Related	No sex and gender related	I don't know
Age				
Genetics				
Sex Hormones				
Reproductive status				
Marital Status				
Ethnicity				
Personality Traits				
Body Composition				
Religion				
Diet				
Smoking Habit				
Alcohol				
Sexual Orientation				
Environment				
Working Status				
Body size				
Geographic Location				
Disability				
Co-Morbidities				
Socioeconomic Status				

At the best of your knowledge, in randomized control trials to register new drugs which is the average percentage of women enrollment? *

- <10%
- 10-30%
- 31-50%
- 51-70%
- >70%

Have you ever read in clinical guidelines if any recommendation is proposed specifically for men or women? *

- Yes
- No

If Yes, which guideline you refer to? (If no, please leave this section blank)

La tua risposta

Among the following topics which are the ones you are most interested in knowing if sex and gender differences exist and influence the clinical management? (please max 3 answers) *

- Cardiovascular Diseases
- Vascular disease other than cardiac ones
- Intestinal Bowel Diseases
- Kidney Diseases
- Cerebral Diseases/Cognitive Diseases
- Lung Diseases
- Infectious Diseases
- Immunological Diseases
- Rheumatological Diseases
- Liver Diseases
- Blood Diseases
- Metabolic diseases
